# NF90 in Posttranscriptional Gene Regulation and MicroRNA Biogenesis

**DOI:** 10.3390/ijms140817111

**Published:** 2013-08-19

**Authors:** Kiyoshi Masuda, Yuki Kuwano, Kensei Nishida, Kazuhito Rokutan, Issei Imoto

**Affiliations:** 1 Department of Human Genetics and Public Health, Institute of Health Biosciences, the University of Tokushima Graduate School, Tokushima 770-8503, Japan; E-Mail: issehgen@tokushima-u.ac.jp; 2 Department of Stress Science, Institute of Health Biosciences, the University of Tokushima Graduate School, Tokushima 770-8503, Japan; E-Mails: kuwanoy@tokushima-u.ac.jp (Y.K.); knishida@tokushima-u.ac.jp (K.N.); rokutan@tokushima-u.ac.jp (K.R.)

**Keywords:** NF90, double-stranded RNA binding proteins, posttranscriptional regulation, microRNA biogenesis

## Abstract

Gene expression patterns are effectively regulated by turnover and translation regulatory (TTR) RNA-binding proteins (RBPs). The TTR-RBPs control gene expression at posttranscriptional levels, such as pre-mRNA splicing, mRNA cytoplasmic export, turnover, storage, and translation. Double-stranded RNA binding proteins (DSRBPs) are known to regulate many processes of cellular metabolism, including transcriptional control, translational control, mRNA processing and localization. Nuclear factor 90 (NF90), one of the DSRBPs, is abundantly expressed in vertebrate tissue and participates in many aspects of RNA metabolism. NF90 was originally purified as a component of a DNA binding complex which binds to the antigen recognition response element 2 in the interleukin 2 promoter. Recent studies have provided us with interesting insights into its possible physiological roles in RNA metabolism, including transcription, degradation, and translation. In addition, it was shown that NF90 regulates microRNA expression. In this review, we try to focus on the function of NF90 in posttranscriptional gene regulation and microRNA biogenesis.

## 1. Introduction

Posttranscriptional gene regulation contributes to biological diversity in many physiological activities and pathological conditions, such as tissue specificity, aging, oxidative stress, inflammation and tumorigenesis. The turnover and translation regulatory (TTR) RNA-binding proteins (RBPs) and non-coding RNAs including microRNAs (miRNAs), influence many posttranscriptional processes, such as pre-mRNA splicing, transport, degradation, storage and translation [1,2]. Although some TTR-RBPs regulate a specific posttranscriptional process, e.g., tristetraprolin (TTP) and KH-type splicing regulatory protein (KSRP) specifically promote mRNA degradation [3–5], most TTR-RBPs can modulate multiple posttranscriptional processes. For example, HuR and nuclear factor 90 (NF90) regulate both mRNA translation and stability [6–9]. These TTR-RBPs regulate the posttranscriptional fate of target mRNAs, mainly by binding to their target sequences within the 3′-untranslated region (UTR), but sometimes within the 5′UTR or the coding region [10–12].

A group of double-stranded (ds) RNA binding proteins (DSRBPs) contain a nucleic acid-binding motif (double-stranded RNA-binding motif, dsRBM), which characteristically binds to A-form double helix RNAs and several highly structured single-stranded (ss) RNAs, such as the adenovirus VA RNAs, but not to dsDNA, ssDNA, or ssRNA [13]. DSRBPs contribute to several aspects of cellular metabolism, including transcriptional activation, translational control, and mRNA processing and localization. Since most dsRBM-dsRNA interactions are mediated by phosphodiester backbone and 2′-OH groups, dsRBMs do not have any RNA sequence specificity. However, sequence-dependent structures of RNAs could provide a possible source of binding specificity recognized by dsRBMs [14–17]. The dsRBMs also contribute to protein-protein interactions between DSRBPs in the absence of dsRNA [18–20].

Among DSRBPs, the NF90 family harbors interesting abilities to bind to ssRNA at high concentrations, DNA-RNA hybrids, ssDNA and dsDNA [20–24]. The NF90 family is known to be abundantly expressed in vertebrate tissue [25], and to participate in many aspects of RNA metabolism, including transcription, degradation, and translation [26]. Here, we try to focus on the function of this unique DSRBP NF90 family in posttranscriptional gene regulation and miRNA biogenesis.

## 2. NF90 Structure

**Figure 1:**
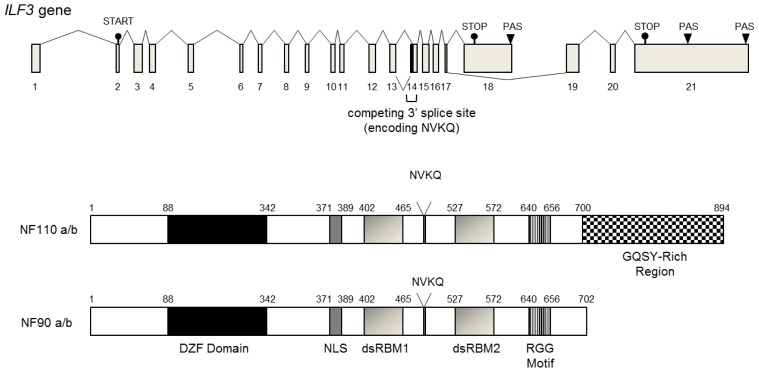
NF90 structure. Diagram of NF90 and NF110 mRNA isoforms. Location of each exon is indicated by Arabic numbers. The translation start site in exon 2 is indicated by START. The translation stop sites in exon 18 and exon 21 are indicated by STOP. Polyadenylation sites are indicated by PAS.

The NF90 family is known to be transcribed from the interleukin enhancer binding factor 3 (ILF3) gene, and at least five distinct transcripts generated by two alternative splicing and three polyadenylation events have been identified in a human melanoma cDNA library (Figure 1) [27]. The two most prominent protein isoforms have apparent molecular masses of 90 kDa and 110 kDa, and are termed NF90 (also known as DRBP76 and NFAR1) and NF110 (also known as ILF3, NFAR2, and TCP110), respectively. Both NF90 and NF110 are homologous in the *N*-terminal and central regions but they vary at their *C*-termini [27–29]. Since an additional alternate splicing event at the competing 3′ splice site of the intron 13/exon 14 boundary inserts four amino acids (NVKQ) into the region between the two dsRBMs, NF90 and NF110 are separated into two further isoforms, NF90a/b and NF110a/b. NF90b and NF110b have the NVKQ insert, while NF90a and NF110a do not [27].

NF90 and NF110 contain a DZF (dsRBM and zinc finger associated) domain in the *N*-terminal regions, a nuclear localization signal (NLS) and two dsRBMs in the central regions, and an RGG motif that is capable of nucleic acid binding in the *C*-terminal region (Figure 1). The DZF domains are conserved in other proteins, such as nuclear factor 45 (NF45), spermatid perinuclear RNA-binding protein (SPNR) and zinc-finger protein associated with RNA (ZFR) [30]. Through the DZF domain, NF45 forms a heterodimer with NF90 or NF110 and stabilizes them [31]. NF110 also has an additional GQSY-rich region that can interact with nucleic acids.

## 3. NF90 Function in Transcription

NF90 was originally identified as a component of a DNA binding complex that interacts with the antigen recognition response element 2 (ARRE-2) in the interleukin 2 (IL-2) promoter and participates in the up-regulation of IL-2, which is a cytokine required for T-cell proliferation [22]. Several lines of evidence have shown that NF90 and NF45 could function as DNA-binding proteins [24,32,33], although they do not contain any DNA-binding motifs and the NF90/NF45 complex does not have any direct ability to interact with DNA. Recent reports showed that NF90 and NF45 are in a complex containing the Ku proteins, DNA-protein kinase (PK) and eukaryotic initiation factor 2 (eIF2), suggesting that the interaction between the NF90/NF45 complex and DNA is mediated by these protein partners [34,35]. Furthermore, NF90 also interacts with the dsRNA-activated protein kinase, PKR, and acts as a substrate as well as a regulator of PKR [20,25,36,37].

NF90 is predominantly expressed in the nucleus, and can regulate transcription in mammalian cells either positively or negatively [25,26,38]. The NF90/NF45 complex stimulates transcription from a cytomegalovirus (CMV) promoter. The association of NF45 with NF90 induces an active conformation of NF90, which is capable of being recruited to a CMV promoter region [26]. NF90 also inhibits transcription from the adenovirus ML1 promoter [26]. NF90 is recruited to the Gal4 binding sites of the ML1 promoter by the *S.cerevisiae* Gal4 binding domain, independently of the association with NF45. The *Xenopus* homologs of NF110 and NF90, CBTF122 and CBTF98 respectively, form the CAATT-box transcription factor complex and bind to the CAATT regulatory element in the GATA-2 promoter after the mid-blastula transition. This complex is sequestered in the cytoplasm by binding to the untranslated mRNAs, and translocated into the nucleus when these RNAs are degraded in the cytoplasm. The dsRBMs of CBTF122 are essential for this regulatory control [23,39].

## 4. NF90 in Post-Transcriptional Gene Regulation

Although NF90 is mostly expressed in the nucleus, several stimuli are able to cause its translocation to the cytoplasm. Recent transcriptome analyses including ribonucleoprotein immunoprecipitation followed by microarray analysis (RIP-chip) have revealed dozens of NF90 targets in the cytoplasm. Sequence comparison of NF90 target mRNAs revealed that NF90-associated mRNAs mostly contain a 25- to 30-nucleotide-long, highly AU-rich motif in their 3′UTR [40]. Neplioueva *et al.* also reported tissue-specific expression of NF90, and identified its target mRNAs in HEK293 cells [41]. These results indicate that NF90 could contribute to posttranscriptional gene regulation.

### 4.1. Control of mRNA Turnover

The half-life of an mRNA varies in response to different stimuli. Since the stability of mRNA decreases or increases rapidly, the levels of mRNA in the cell can change dramatically without transcriptional changes [42,43]. The half-life of mRNA is usually regulated by AU-rich elements located within its 3′UTR [44]. NF90 was shown to enhance the stability of many transcripts in response to several stimuli (Table 1). Shim and co-workers showed that NF90 translocates from the nucleus to the cytoplasm during T-cell activation [7]. Cytoplasmic NF90 bound to AU-rich elements in the 3′UTR of *IL-2* mRNA, and inhibited its degradation. Oxidative stress by hydrogen peroxide increases the association of NF90 and HuR to the 3′UTR of the *mitogen-activated protein kinase phosphatase 1* (*MKP-1*) mRNA, and stabilizes it [45]. Since NF90 can also inhibit *MKP-1* translation, NF90 dissociates from *MKP-1* mRNA by 4 h after exposure to the oxidant, sparing *MKP-1* mRNA from translational suppression. In response to hypoxia, NF90 binds to the stem-loop hypoxia stability region in the 3′UTR of the *vascular endothelial growth factor* (*VEGF*) mRNA, and increases its stability without altering the HIF-1-dependent transcriptional activity [46]. The cyclin-dependent kinase inhibitor *p21^WAF1/Cip1^* and the myogenic transcription factor *MyoD* mRNA are also targets of NF90, since these mRNAs contain secondary structures that include regions of dsRNA and hairpin loops. NF90 stabilizes these mRNAs and promotes myogenic differentiation of skeletal muscles [47].

The mechanisms by which NF90 stabilizes its target mRNAs remain largely unknown. CD28 stimulates AKT-mediated phosphorylation of NF90 at Ser^647^, which induced NF90 translocation to the cytoplasm, leading to stabilization of *IL-2* mRNA [48]. PMA stimulation of Jurkat T cells also promotes PKCβ1-mediated phosphorylation of NF90 at Ser^647^ and induces nuclear export of NF90 and *IL-2* mRNA stabilization [49]. The association of NF90 to target mRNA might block the assembly of destabilizing RBPs and/or microRNAs onto their target mRNAs, as recently shown for HuR [50]. Recently, the long noncoding RNA-Low Expression in Tumor (lncRNA-LET), was shown to interact with the NF90 protein. The association of lncRNA-LET with NF90 promotes the NF90 protein ubiquitination and degradation, leading to destabilization of target mRNAs [51].

### 4.2. Control of mRNA Translation

The translation of mRNA is regulated at the initiation (by increasing or decreasing the loading of ribosomes onto the mRNA), the elongation, or the termination steps. NF90 has been shown to regulate translation of some target mRNAs (Table 1). NF90 inhibits the translation of *β-glucosidase*, *MKP-1*, *MCP-1*, *GROa*, *IL-6*, and *IL-8* mRNAs [28,45,52]. Recently, RIP-chip analysis revealed that NF90 represses the translation of *CCNA*, *CCNI*, *CDC2*, and *EIF4E* mRNAs, which contain the predicted NF90 binding motif (UAAUGAAUUAUUUAUCUUAUUUAA) in their 3′UTRs [40]. In contrast, NF90 activates the translation of *VEGF* mRNA in response to hypoxia and *cyclin T1* in HIV-1 infection [44,53].

The crucial mechanism whereby NF90 regulates translation is also still unknown. Kuwano *et al.* showed that NF90 competed with the translational repressors TIAR and TIA-1 for binding to *MKP-1* mRNAs in response to oxidative stress [45]. Pfeifer *et al.* showed that NF90 functions as a general inhibitor of mRNA export to the cytoplasm and depletion of NF90 causes an increase in protein synthesis rate [54]. It also could be hypothesized that binding of NF90 blocks the association of the translation initiation factors on target mRNA. Moreover, NF90 might recruit gene silencing factors, such as microRNAs, DICER, PACT and TRBP, onto the target mRNA [55–57]. In contrast, NF90 activates translation of *cyclin T1* mRNA, facilitating 5′–3′ loop formation via interaction with eIF2, ribosomes, and ribosomal protein S19 [53]. Further study is needed to fully elucidate the precise molecular mechanisms of NF90-mediated translational regulation.

## 5. NF90 in MicroRNA Biogenesis

The miRNAs are a class of ~21-nucleotide-long RNAs that regulate gene expression by degradation of RNA transcripts or translational inhibition of target mRNAs [58]. In mammals, less than 50% of protein-coding genes are regulated by miRNAs. Generally, miRNAs are transcribed from their genes as primary miRNA transcripts (pri-miRNA) by RNA polymerase II [59]. Then, Microprocessor complex containing Drosha and DGCR8 processes these pri-miRNAs into precursor miRNAs (pre-miRNAs). After this processing, the pre-miRNAs are exported to the cytoplasm by Exportin-5 and RanGTP [60] and cleaved into 22-nucleotide-long miRNA/miRNA* duplexes by Dicer [56]. One strand of this duplex, representing a mature miRNA, is then loaded into the miRNA-induced silencing complex (miRISC) which contains Argonaute (AGO) protein and glycine-tryptophan protein of 182 kDa (GW182), leading to interaction with target mRNAs that induces their translational repression, or degradation [58,61].

The Microprocessor complex forms a large protein complex with NF90 and NF45 [62]. Recently, it was shown that this NF90/NF45 protein complex functions as a negative regulator in miRNA biogenesis. Overexpression of NF90 and NF45 inhibits the production of miRNAs, including let-7a, miR-21, miR-185, miR-23b, miR-34a, miR-193a, miR-193b, and miR-181a, by blocking the processing of pri-miRNA [63–65]. In contrast, NF90/NF45 complex elevates the expression of miR-193b, miR-196b and miR-621 but decreases their pre-miRNAs levels, indicating that these miRNAs are regulated by a different processing pathway [64].

The molecular roles of NF90 and NF45 in miRNA biogenesis have never been fully elucidated. Sakamoto *et al.* indicated that the NF90/NF45 complex might compete for the association of the Microprocessor complex with pri-miRNAs, leading to a decrease in miRNA production [63].

## 6. NF90 Functions in Cellular Processes and Diseases

It has been suggested that NF90 is involved in many cellular processes, including immunity, differentiation, cell proliferation, angiogenesis, and the cell cycle [7,46,47,66,67]. Depletion of NF90 or NF45 suppresses cell growth by inhibiting DNA synthesis in HeLa cells [31]. In human tissues, the NF90 family proteins and NF45 are highly expressed in brain, testis, and thymus, whereas they are minimally expressed in lung, spleen, skeletal muscle, and liver, even though these mRNAs are ubiquitously expressed [25,47,68,69]. These expression profiles suggest that these proteins could be more highly expressed in undifferentiated compared to differentiated cells.

NF90 also regulates mRNAs encoded by senescence-related genes. It has been implied that NF90 contributes to suppressing the acquisition of a senescence-associated secretory phenotype (SASP). In proliferating human fibroblasts, NF90 is highly expressed and inhibits the expression of SASP mRNAs by reducing their translation. In contrast, NF90 protein expression is markedly reduced in senescent fibroblasts. This reduction stimulates expression of numerous SASP factors, such as MCP-1, GROa, IL-6, and IL-8 [52].

Cancer is one of the most prominent age-related diseases, and NF90 is increasingly recognized as a crucial regulator of cancer-related gene expression. Overexpression of NF90 was reported in nasopharyngeal carcinoma [66], non-small cell lung carcinoma [70], hepatocarcinoma [63], ovarian cancer [71], and breast cancer [65]. In breast cancer tissues, nuclear NF90 expression, but not cytoplasmic NF90 expression, correlates with urokinase-type plasminogen activator (uPA) levels and clinical tumor grades [65]. Moreover, depression of NF90 results in an increase of let-7a miRNA, which is a member of the tumor suppressing miRNAs, and growth inhibition in transformed cells [63]. Elucidation of the roles of NF90 in cancer development may reveal possible therapeutic targets for this fatal disease.

## 7. Conclusions and Perspective

In closing, our knowledge of posttranscriptional gene regulation in all biological areas has advanced remarkably over the past two decades. Many genes implicated in cancer, aging and age-related conditions encode mRNAs that are regulated through their stability and/or translation by TTR-RBPs, such as HuR, AUF1, TIA-1, and TTP [72,73]. Since NF90 also functions as a regulator of mRNA transport, stability and translation, systematic analysis of NF90 targeted mRNAs and its interaction with these transcripts is informative for understanding the molecular mechanisms of TTR-RBP-mediated gene expression. As discussed above, NF90 regulates mRNAs that contain AU-rich elements (AREs) in their 3′UTRs. Many other TTR-RBPs, such as HuR, AUF1, TTP, BRF1, and KSRP, can also interact with the mRNAs containing AREs. Therefore, NF90 complexes formed with mRNAs bearing this motif may determine the posttranscriptional fate of target transcripts via competitive or cooperative interaction with other TTR-RBPs. Moreover, association of NF90 with its target mRNAs may be regulated by posttranslational modification of NF90, like NF90 phosphorylation at Ser^647^ [48,49]. Future investigation of these posttranslational modifications and their influence on the metabolism of target mRNAs will provide crucial insight into the function of NF90 as a gene expression regulator.

## Figures and Tables

**Table 1:** Target mRNAs of NF90. The table includes the NF90 target mRNAs (column 1) and the region(s) with which NF90 interacts (column 2), the posttranscriptional consequences of these interactions (column 3), and the conditions that modulate their association with mRNAs (column 4).

Target mRNA	Binding sites	Consequences to mRNA	Conditions	References
β-glucosidase	3′UTR	Translation ↓	-	[28]
IL-2	3′UTR	Stability ↑	T-cell activation	[7]
VEGF	3′UTR	Stability ↑ Translation ↑	Hypoxia	[46]
MKP-1	3′UTR	Stability ↑ Translation ↓	Hydrogen peroxide	[45]
p21^WAF1/Cip1^, MyoD	3′UTR	Stability ↑	-	[47]
CCNA, CCNI, CDC2, EIF4E	3′UTR	Translation ↓	-	[40]
GM-CFS, MCP-1, GROa, IL-6, IL-8	3′UTR	Translation ↓	Proliferating cells	[52]
Cyclin T1	3′UTR	Translation ↑	HIV-1 infection	[53]

## References

[1] Moore M.J. (2005). From birth to death: The complex lives of eukaryotic mRNAs. Science.

[2] Glisovic T., Bachorik J.L., Yong J., Dreyfuss G. (2008). RNA-binding proteins and post-transcriptional gene regulation. FEBS Lett.

[3] Carballo E., Lai W.S., Blackshear P.J. (1998). Feedback inhibition of macrophage tumor necrosis factor-alpha production by tristetraprolin. Science.

[4] Chen C.Y., Gherzi R., Ong S.E., Chan E.L., Raijmakers R., Pruijn G.J., Stoecklin G., Moroni C., Mann M., Karin M. (2001). Au binding proteins recruit the exosome to degrade are-containing mRNAs. Cell.

[5] Briata P., Forcales S.V., Ponassi M., Corte G., Chen C.Y., Karin M., Puri P.L., Gherzi R. (2005). P38-dependent phosphorylation of the mRNA decay-promoting factor KSRP controls the stability of select myogenic transcripts. Mol. Cell.

[6] Gorospe M. (2003). Hur in the mammalian genotoxic response: Post-transcriptional multitasking. Cell Cycle.

[7] Shim J., Lim H., Yates J.R., Karin M. (2002). Nuclear export of NF90 is required for interleukin-2 mRNA stabilization. Mol. Cell.

[8] Xu Y.H., Busald C., Grabowski G.A. (2000). Reconstitution of TCP80/NF90 translation inhibition activity in insect cells. Mol. Genet. Metab.

[9] Xu Y.H., Leonova T., Grabowski G.A. (2003). Cell cycle dependent intracellular distribution of two spliced isoforms of TCP/ILF3 proteins. Mol. Genet. Metab.

[10] Mitchell P., Tollervey D. (2000). mRNA stability in eukaryotes. Curr. Opin. Genet. Dev.

[11] Orphanides G., Reinberg D. (2002). A unified theory of gene expression. Cell.

[12] Masuda K., Abdelmohsen K., Gorospe M. (2009). RNA-binding proteins implicated in the hypoxic response. J. Cell Mol. Med.

[13] Fierro-Monti I., Mathews M.B. (2000). Proteins binding to duplexed RNA: One motif, multiple functions. Trends Biochem. Sci.

[14] Bycroft M., Grunert S., Murzin A.G., Proctor M., St Johnston D. (1995). NMR solution structure of a dsRNA binding domain from *Drosophila staufen* protein reveals homology to the *N*-terminal domain of ribosomal protein S5. EMBO J.

[15] Kharrat A., Macias M.J., Gibson T.J., Nilges M., Pastore A. (1995). Structure of the dsRNA binding domain of *E. coli* RNAse iii. EMBO J.

[16] Bevilacqua P.C., Cech T.R. (1996). Minor-groove recognition of double-stranded RNA by the double-stranded RNA-binding domain from the RNA-activated protein kinase PKR. Biochemistry.

[17] Ryter J.M., Schultz S.C. (1998). Molecular basis of double-stranded RNA-protein interactions: Structure of a dsRNA-binding domain complexed with dsRNA. EMBO J.

[18] Cosentino G.P., Venkatesan S., Serluca F.C., Green S.R., Mathews M.B., Sonenberg N. (1995). Double-stranded-RNA-dependent protein kinase and TAR RNA-binding protein form homo- and heterodimers *in vivo*. Proc. Natl. Acad. Sci. USA.

[19] Patel R.C., Stanton P., McMillan N.M., Williams B.R., Sen G.C. (1995). The interferon-inducible double-stranded RNA-activated protein kinase self-associates *in vitro* and *in vivo*. Proc. Natl. Acad. Sci. USA.

[20] Parker L.M., Fierro-Monti I., Mathews M.B. (2001). Nuclear factor 90 is a substrate and regulator of the eukaryotic initiation factor 2 kinase double-stranded RNA-activated protein kinase. J. Biol. Chem.

[21] Bass B.L., Hurst S.R., Singer J.D. (1994). Binding properties of newly identified xenopus proteins containing dsRNA-binding motifs. Curr. Biol.

[22] Corthesy B., Kao P.N. (1994). Purification by DNA affinity chromatography of two polypeptides that contact the NF-AT DNA binding site in the interleukin 2 promoter. J. Biol. Chem.

[23] Orford R.L., Robinson C., Haydon J.M., Patient R.K., Guille M.J. (1998). The maternal CCAAT box transcription factor which controls GATA-2 expression is novel and developmentally regulated and contains a double-stranded-RNA-binding subunit. Mol. Cell Biol.

[24] Satoh M., Shaheen V.M., Kao P.N., Okano T., Shaw M., Yoshida H., Richards H.B., Reeves W.H. (1999). Autoantibodies define a family of proteins with conserved double-stranded RNA-binding domains as well as DNA binding activity. J. Biol. Chem.

[25] Saunders L.R., Perkins D.J., Balachandran S., Michaels R., Ford R., Mayeda A., Barber G.N. (2001). Characterization of two evolutionarily conserved, alternatively spliced nuclear phosphoproteins, NFAR-1 and -2, that function in mRNA processing and interact with the double-stranded RNA-dependent protein kinase, PKR. J. Biol Chem.

[26] Reichman T.W., Muniz L.C., Mathews M.B. (2002). The RNA binding protein nuclear factor 90 functions as both a positive and negative regulator of gene expression in mammalian cells. Mol. Cell Biol.

[27] Duchange N., Pidoux J., Camus E., Sauvaget D. (2000). Alternative splicing in the human interleukin enhancer binding factor 3 (ILF3) gene. Gene.

[28] Xu Y.H., Grabowski G.A. (1999). Molecular cloning and characterization of a translational inhibitory protein that binds to coding sequences of human acid beta-glucosidase and other mRNAs. Mol. Genet. Metab.

[29] Saunders L.R., Jurecic V., Barber G.N. (2001). The 90- and 110-kda human NFAR proteins are translated from two differentially spliced mRNAs encoded on chromosome 19p13. Genomics.

[30] Wolkowicz U.M., Cook A.G. (2012). NF45 dimerizes with NF90, zfr and spnr via a conserved domain that has a nucleotidyltransferase fold. Nucleic Acids Res.

[31] Guan D., Altan-Bonnet N., Parrott A.M., Arrigo C.J., Li Q., Khaleduzzaman M., Li H., Lee C.G., Pe’ery T., Mathews M.B. (2008). Nuclear factor 45 (NF45) is a regulatory subunit of complexes with NF90/110 involved in mitotic control. Mol. Cell Biol.

[32] Ranpura S.A., Deshmukh U., Reddi P.P. (2008). NF45 and NF90 in murine seminiferous epithelium: Potential role in sp-10 gene transcription. J. Androl.

[33] Kao P.N., Chen L., Brock G., Ng J., Kenny J., Smith A.J., Corthesy B. (1994). Cloning and expression of cyclosporin a- and fk506-sensitive nuclear factor of activated t-cells: NF45 and NF90. J. Biol. Chem.

[34] Ting N.S., Kao P.N., Chan D.W., Lintott L.G., Lees-Miller S.P. (1998). DNA-dependent protein kinase interacts with antigen receptor response element binding proteins NF90 and NF45. J. Biol. Chem.

[35] Burckstummer T., Bennett K.L., Preradovic A., Schutze G., Hantschel O., Superti-Furga G., Bauch A. (2006). An efficient tandem affinity purification procedure for interaction proteomics in mammalian cells. Nat. Methods.

[36] Langland J.O., Kao P.N., Jacobs B.L. (1999). Nuclear factor-90 of activated t-cells: A double-stranded RNA-binding protein and substrate for the double-stranded RNA-dependent protein kinase, PKR. Biochemistry.

[37] Coolidge C.J., Patton J.G. (2000). A new double-stranded RNA-binding protein that interacts with PKR. Nucleic Acids Res.

[38] Krasnoselskaya-Riz I., Spruill A., Chen Y.W., Schuster D., Teslovich T., Baker C., Kumar A., Stephan D.A. (2002). Nuclear factor 90 mediates activation of the cellular antiviral expression cascade. AIDS Res. Hum. Retroviruses.

[39] Brzostowski J., Robinson C., Orford R., Elgar S., Scarlett G., Peterkin T., Malartre M., Kneale G., Wormington M., Guille M. (2000). RNA-dependent cytoplasmic anchoring of a transcription factor subunit during xenopus development. EMBO J.

[40] Kuwano Y., Pullmann R., Marasa B.S., Abdelmohsen K., Lee E.K., Yang X., Martindale J.L., Zhan M., Gorospe M. (2010). NF90 selectively represses the translation of target mRNAs bearing an AU-rich signature motif. Nucleic Acids Res..

[41] Neplioueva V., Dobrikova E.Y., Mukherjee N., Keene J.D., Gromeier M. (2010). Tissue type-specific expression of the dsRNA-binding protein 76 and genome-wide elucidation of its target mRNAs. PLoS One.

[42] Mitchell P., Tollervey D. (2001). mRNA turnover. Curr. Opin. Cell Biol.

[43] Wilusz C.J., Wormington M., Peltz S.W. (2001). The cap-to-tail guide to mRNA turnover. Nat. Rev. Mol. Cell Biol.

[44] Chen C.Y., Shyu A.B. (1995). AU-rich elements: Characterization and importance in mRNA degradation. Trends Biochem. Sci.

[45] Kuwano Y., Kim H.H., Abdelmohsen K., Pullmann R., Martindale J.L., Yang X., Gorospe M. (2008). MKP-1 mRNA stabilization and translational control by RNA-binding proteins HuR and NF90. Mol. Cell Biol..

[46] Vumbaca F., Phoenix K.N., Rodriguez-Pinto D., Han D.K., Claffey K.P. (2008). Double-stranded RNA-binding protein regulates vascular endothelial growth factor mRNA stability, translation, and breast cancer angiogenesis. Mol. Cell Biol.

[47] Shi L., Zhao G., Qiu D., Godfrey W.R., Vogel H., Rando T.A., Hu H., Kao P.N. (2005). NF90 regulates cell cycle exit and terminal myogenic differentiation by direct binding to the 3′-untranslated region of MyoD and p21WAF1/CIP1 mRNAs. J. Biol. Chem.

[48] Pei Y., Zhu P., Dang Y., Wu J., Yang X., Wan B., Liu J.O., Yi Q., Yu L. (2008). Nuclear export of NF90 to stabilize IL-2 mRNA is mediated by AKT-dependent phosphorylation at Ser^647^ in response to CD28 costimulation. J. Immunol.

[49] Zhu P., Jiang W., Cao L., Yu W., Pei Y., Yang X., Wan B., Liu J.O., Yi Q., Yu L. (2010). IL-2 mRNA stabilization upon PMA stimulation is dependent on NF90-Ser^647^ phosphorylation by protein kinase CbetaI. J. Immunol.

[50] Hinman M.N., Lou H. (2008). Diverse molecular functions of Hu proteins. Cell Mol. Life Sci.

[51] Yang F., Huo X.S., Yuan S.X., Zhang L., Zhou W.P., Wang F., Sun S.H. (2013). Repression of the long noncoding RNA-let by histone deacetylase 3 contributes to hypoxia-mediated metastasis. Mol. Cell.

[52] Tominaga-Yamanaka K., Abdelmohsen K., Martindale J.L., Yang X., Taub D.D., Gorospe M. (2012). NF90 coordinately represses the senescence-associated secretory phenotype. Aging.

[53] Hoque M., Shamanna R.A., Guan D., Pe’ery T., Mathews M.B. (2011). HIV-1 replication and latency are regulated by translational control of cyclin T1. J. Mol. Biol.

[54] Pfeifer I., Elsby R., Fernandez M., Faria P.A., Nussenzveig D.R., Lossos I.S., Fontoura B.M., Martin W.D., Barber G.N. (2008). NFAR-1 and -2 modulate translation and are required for efficient host defense. Proc. Natl. Acad. Sci. USA.

[55] Kim H.H., Kuwano Y., Srikantan S., Lee E.K., Martindale J.L., Gorospe M. (2009). HuR recruits let-7/RISC to repress c-Myc expression. Genes Dev.

[56] Chendrimada T.P., Gregory R.I., Kumaraswamy E., Norman J., Cooch N., Nishikura K., Shiekhattar R. (2005). TRBP recruits the Dicer complex to Ago2 for microRNA processing and gene silencing. Nature.

[57] Lee Y., Hur I., Park S.Y., Kim Y.K., Suh M.R., Kim V.N. (2006). The role of pact in the RNA silencing pathway. EMBO J.

[58] Bartel D.P. (2004). MicroRNAs: Genomics, biogenesis, mechanism, and function. Cell.

[59] Lee Y., Kim M., Han J., Yeom K.H., Lee S., Baek S.H., Kim V.N. (2004). MicroRNA genes are transcribed by RNA polymerase II. EMBO J.

[60] Yi R., Qin Y., Macara I.G., Cullen B.R. (2003). Exportin-5 mediates the nuclear export of pre-microRNAs and short hairpin RNAs. Genes Dev.

[61] Krol J., Loedige I., Filipowicz W. (2010). The widespread regulation of microRNA biogenesis, function and decay. Nat. Rev. Genet.

[62] Gregory R.I., Yan K.P., Amuthan G., Chendrimada T., Doratotaj B., Cooch N., Shiekhattar R. (2004). The microprocessor complex mediates the genesis of microRNAs. Nature.

[63] Sakamoto S., Aoki K., Higuchi T., Todaka H., Morisawa K., Tamaki N., Hatano E., Fukushima A., Taniguchi T., Agata Y. (2009). The NF90-NF45 complex functions as a negative regulator in the microRNA processing pathway. Mol. Cell Biol.

[64] Volk N., Shomron N. (2011). Versatility of microRNA biogenesis. PLoS One.

[65] Hu Q., Lu Y.Y., Noh H., Hong S., Dong Z., Ding H.F., Su S.B., Huang S. (2012). Interleukin enhancer-binding factor 3 promotes breast tumor progression by regulating sustained urokinase-type plasminogen activator expression. Oncogene.

[66] Fung L.F., Lo A.K., Yuen P.W., Liu Y., Wang X.H., Tsao S.W. (2000). Differential gene expression in nasopharyngeal carcinoma cells. Life Sci.

[67] Matsumoto-Taniura N., Pirollet F., Monroe R., Gerace L., Westendorf J.M. (1996). Identification of novel M phase phosphoproteins by expression cloning. Mol. Biol. Cell.

[68] Sakamoto S., Morisawa K., Ota K., Nie J., Taniguchi T. (1999). A binding protein to the DNase I hypersensitive site II in HLA-DR alpha gene was identified as NF90. Biochemistry.

[69] Zhao G., Shi L., Qiu D., Hu H., Kao P.N. (2005). NF45/ILF2 tissue expression, promoter analysis, and interleukin-2 transactivating function. Exp. Cell Res.

[70] Guo N.L., Wan Y.W., Tosun K., Lin H., Msiska Z., Flynn D.C., Remick S.C., Vallyathan V., Dowlati A., Shi X. (2008). Confirmation of gene expression-based prediction of survival in non-small cell lung cancer. Clin. Cancer Res.

[71] Guo Y., Fu P., Zhu H., Reed E., Remick S.C., Petros W., Mueller M.D., Yu J.J. (2012). Correlations among ercc1, XPB, UBE2I, EGF, TAL2 and ILF3 revealed by gene signatures of histological subtypes of patients with epithelial ovarian cancer. Oncol. Rep.

[72] Abdelmohsen K., Gorospe M. (2010). Posttranscriptional regulation of cancer traits by HuR. Wiley Interdiscip. Rev. RNA.

[73] Masuda K., Kuwano Y., Nishida K., Rokutan K. (2012). General RBP expression in human tissues as a function of age. Ageing Res. Rev.

